# Congenital anterior urethrocutaneous fistula: a single-center retrospective study

**DOI:** 10.3389/fsurg.2025.1527649

**Published:** 2025-06-17

**Authors:** Chao Yang, Chi Zhang, Yongsheng Cao, Xin Yu

**Affiliations:** ^1^ Department of Urology, Anhui Provincial Children's Hospital, Hefei, Anhui, China; ^2^ Department of Oncology, Anhui Chest Hospital, Hefei, Anhui, China

**Keywords:** congenital, anterior urethra, urethrocutaneous fistula, penis, hypospadias

## Abstract

**Background:**

This study aims to investigate the clinical characteristics and treatment strategies for congenital anterior urethrocutaneous fistula.

**Methods:**

We retrospectively analyzed the clinical data of patients with congenital anterior urethrocutaneous fistula who underwent surgical treatment at Anhui Children's Hospital from December 2009 to February 2023. Data collected included patient demographics, preoperative fistula characteristics, surgical details, and postoperative outcomes. Postoperative follow-up was conducted regularly to evaluate clinical outcomes.

**Results:**

The average age of the eight patients was 31.38 ± 18.70 months. Fistula locations included three at the coronal sulcus, three at the penile midshaft, one at the penoscrotal junction, and one on the scrotum. The mean length of the fistulas was 0.66 ± 0.28 cm. Two patients presented with associated penile curvature and scrotal raphe splitting, while one had isolated penile curvature. Seven patients underwent primary repair: for cases with fistulas at the coronal sulcus (Cases 2 and 8), the Mathieu technique was utilized due to well-developed glans and sufficient ventral subcutaneous tissue; three patients (Cases 1, 3, and 5) with well-developed urethral plates underwent the Duplay technique; for two cases (Cases 4 and 7) with narrow urethral plates, the TIP and Onlay techniques were employed, respectively. In Case 6, due to severe penile curvature, a staged approach was necessary, involving transection of the urethral plate with initial Duckett procedure and proximal urethrostomy, followed by urethrostomy closure as a secondary procedure. The mean duration of the first-stage surgery for all 8 patients was 100.75 ± 27.88 min. The urinary catheters were removed 12–14 days postoperatively for all surgeries. During a follow-up period of 15–154 months, all patients exhibited normal urination with no evidence of urethral fistula, stricture, diverticulum, or recurrent penile curvature.

**Conclusions:**

The surgical outcomes of congenital anterior urethrocutaneous fistula are generally favorable. For patients without penile curvature, with well-developed urethral plates and sufficient surrounding fascial tissue, the Mathieu or Duplay techniques are suitable for repair. The Onlay or TIP techniques may be preferred for those with narrow urethral plates. For cases with severe penile curvature, transection of the urethral plate with staged repair may be warranted.

## 1 Introduction

Congenital anterior urethrocutaneous fistula is a rare anomaly in urethral development, characterized by a congenital fistula on the ventral penile shaft, with an intact distal urethra and corpus spongiosum. The condition is defined by the coexistence of a fistula opening and urethral meatus within the anterior urethra (1–3). Fistulas can appear at various sites along the anterior urethra, with the urethral meatus either in the normal position or ectopically located between the glans penis and the fistula opening (3, 4).

Congenital urethrocutaneous fistula can be classified into three types, with rectal-urethral fistula being the most common, followed by posterior urethral fistula, and congenital anterior urethrocutaneous fistula being exceedingly rare (5, 6). Gupta first reported this condition in 1962 (7), and to date, fewer than 40 related cases have been published, most of which are case reports. Unlike secondary fistulas resulting from hypospadias surgery or trauma (3, 8), congenital anterior urethrocutaneous fistula arises from intrinsic developmental abnormalities of the urethra itself. It may be associated with ventral preputial defects, penile curvature, hypospadias, redundant urethra, and anorectal malformations. The fistula may occur at any location on the ventral aspect of the penis and must be differentiated from Y-shaped urethral duplication and hypospadias (6, 9). Small fistulas may be as small as a pinhole, while more severe cases can involve the entire penile urethra (6, 8, 10).

Clinical presentations vary according to fistula type and may occur independently or alongside other malformations, such as hypospadias, penile curvature, or anorectal atresia, necessitating tailored surgical approaches.

## 2 Materials and methods

### 2.1 Study design

This retrospective study was approved by our institution's Ethics Committee (Approval No.: EYLL-2024-079) and conducted in accordance with the Declaration of Helsinki (2013 revision). Written informed consent was obtained from the legal guardians of all eligible patients. A total of eight cases of congenital anterior urethrocutaneous fistula underwent surgical treatment at Anhui Children's Hospital between December 2009 and February 2023. The collected data included patient demographics, surgical parameters, and postoperative outcomes (Patient age, age at onset, location of the fistula, length and diameter of the fistula, presence of urethral diverticulum, associated anomalies such as penile chordee or scrotal raphe abnormalities, classification of the urethral fistula, specific surgical techniques used, materials employed for urethral coverage, duration of surgery, duration of catheterization, and postoperative complications). Statistical analyses were conducted using SPSS software version 23.0. Clinical data for these cases were analyzed to examine their characteristics and evaluate surgical strategies.

### 2.2 Inclusion and exclusion criteria of study subjects

Inclusion Criteria: Patients under the age of 18 years with a clinical diagnosis of congenital anterior urethrocutaneous fistula were included in this study. The diagnosis was based on the following: medical history, physical examination findings (presence of a congenital urethral fistula on the ventral surface of the penile shaft, with an intact distal urethra and corpus spongiosum, and coexistence of the fistula opening with the external urethral meatus in the anterior urethra). All included patients underwent surgical intervention.Exclusion Criteria: Acquired urethrocutaneous fistula resulting from trauma, infection, or surgical complications. Coexisting severe urethral anomalies, such as bladder exstrophy, that could interfere with the analysis. Patients with less than 12 months of postoperative follow-up were excluded to ensure that delayed complications, such as fistula recurrence, urethral stricture formation, and long-term cosmetic dissatisfaction, could be adequately assessed.

### 2.3 Surgical techniques

#### 2.3.1 Preoperative interventions

A detailed medical history was obtained, focusing on the presence of anorectal malformations, the location of the fistula, voiding patterns, and associated symptoms. Physical examination was performed to assess the morphology and size of the fistula, as well as its relationship with the external urethral meatus.

Before determining the need for surgical correction, the patency of the distal urethra was evaluated. In complex cases with concomitant anorectal malformations, routine investigations included voiding cystourethrography (VCUG), cystourethroscopy, abdominal ultrasonography, and residual urine volume measurement.

For patients without anorectal malformations, if normal voiding was observed and a catheter could be easily passed through the external urethral meatus into the fistula, VCUG and cystourethroscopy were deemed unnecessary. These procedures often require anesthesia in children and impose additional burdens. In such cases, physical examination was typically sufficient to identify other abnormalities, including urethral patency, the anatomical relationship between the fistula, urethra, and external meatus, as well as the presence of urethral narrowing, diverticulum formation, or abnormalities in the surrounding tissues.

#### 2.3.2 Surgical strategies

All patients underwent surgery under general anesthesia. Initially, the membranous urethra proximal and distal to the fistula was incised up to the healthy urethral spongiosum. The repair method was selected based on the fistula's location and size, any associated anomalies, and the development of the urethra and corpus spongiosum. For anterior urethrocutaneous fistulas located at the coronal sulcus without penile curvature, with a well-developed glans and adequate ventral subcutaneous tissue, the Mathieu technique was utilized. In cases without penile curvature, where the urethral plate was well-developed and surrounded by sufficient fascial tissue, the Duplay technique was employed. For patients with a narrow urethral plate, the Onlay technique or the TIP technique was preferred. In cases with severe penile curvature, the urethral plate was divided, and reconstruction was performed following the principles of hypospadias repair. Soft tissue coverage of the urethra was achieved using periurethral fascia, dartos fascia, or pedicled preputial flaps. Patients with scrotal raphe splitting received concurrent corrective treatment.

#### 2.3.3 Postoperative interventions

An F8 catheter was placed postoperatively to divert urine and prevent leakage through the repaired site, typically retained for 12–14 days. The penis was wrapped with gauze and an elastic bandage. Postoperative external dressings were removed 3–5 days after surgery to evaluate wound healing at the surgical site, with careful observation for signs of exudation, infection, or fistula recurrence. Wound cleanliness was maintained, avoiding exposure to contaminants. Pain management was provided based on the child's pain level, with appropriate analgesics such as acetaminophen or ibuprofen.

After catheter removal, voiding function was observed to ensure smooth urine flow and to monitor for urinary incontinence or retention. Follow-ups were scheduled at 1 month, 3 months, and 1 year postoperatively to evaluate for fistula recurrence, urethral stricture, or diverticulum formation. For patients with postoperative complications (e.g., urethral stricture or fistula recurrence), follow-up evaluations were tailored according to clinical presentation. When urethral stricture or diverticulum was suspected, further diagnostic assessments, including uroflowmetry and VCUG, were performed.

### 2.4 Statistical analysis

Data were analyzed using SPSS software version 23.0. Variables such as age, fistula length, and operation time were continuous variables and were found to follow a normal distribution based on the normality test, represented as mean ± standard deviation (SD). The duration of urinary catheter placement was a continuous variable, and the normality test indicated a non-normal distribution, represented as M(Q1, Q3). Descriptive statistics, including mean, median, and range, were used to summarize patient demographics and surgical outcomes. Given the small sample size, no inferential statistical tests were performed.

## 3 Results

A total of 8 patients were included in this study, with an average age of 31.38 ± 18.70 months. All patients had a normally positioned urethral meatus, intact foreskin, and a normally developed glans, with no associated anorectal abnormalities. Preoperatively, none of the eight patients experienced urinary difficulties or other voiding abnormalities. During preoperative assessment, a catheter could be easily passed through the external urethral meatus into the fistula in all cases. Fistula locations included three cases at the coronal sulcus, three at the penile midshaft, one at the penoscrotal junction, and one on the scrotum. The mean length of the fistulas was 0.66 ± 0.28 cm. Two patients had concomitant penile curvature and scrotal raphe splitting, while one had isolated penile curvature. Seven patients presented with a fistula at birth, while one developed a midshaft penile swelling that bulged with urination and subsequently ruptured, forming a fistula. All fistulas were surrounded by varying degrees of membranous urethral tissue (Table 1).

**Table 1:** Clinical data of 8 cases of congenital anterior urethrocutaneous fistula in children.

NO	Case 1	Case 2	Case 3	Case 4	Case 5	Case 6	Case 7	Case 8
Age(months)	15	55	26	20	30	14	26	65
Fistula location	Midshaft of the penis	Coronal sulcus	Midshaft of the penis	Scrotum	Midshaft of the penis	Junction of the penis and scrotum	Coronal sulcus	Coronal sulcus
Fistula long diameter(cm)	0.3	0.5	1.0	0.8	1.1	0.6	0.6	0.4
Urethral diverticulum	None	None	None	None	Yes	None	None	None
Onset time	After birth	After birth	After birth	After birth	2 months after birth	After birth	After birth	After birth
Associated malformations	None	None	None	Penile curvature downward 30°, scrotal longitudinal cleft	None	Penile curvature downward 35°, scrotal longitudinal cleft	Penile curvature downward 25°	None
Classification	Simple type	Simple type	Simple type	Hypospadias type	Simple type	Hypospadias type	Hypospadias type	Simple type
Surgical method	Duplay	Mathieu	Duplay	TIP	Duplay	Primary Duckett + proximal urethrostomy, secondary stomal closure surgery	Onlay	Mathieu
Covering material	Surrounding fascia	Surrounding fascia	Surrounding fascia	scrotal dartos	Surrounding fascia	scrotal dartos	Surrounding fascia	Surrounding fascia
Primary surgery duration (min)	85	58	104	130	104	135	120	70
Catheter indwelling (days)	12	12	13	14	12	Primary 14, secondary 14	14	12

**Figure 1:**
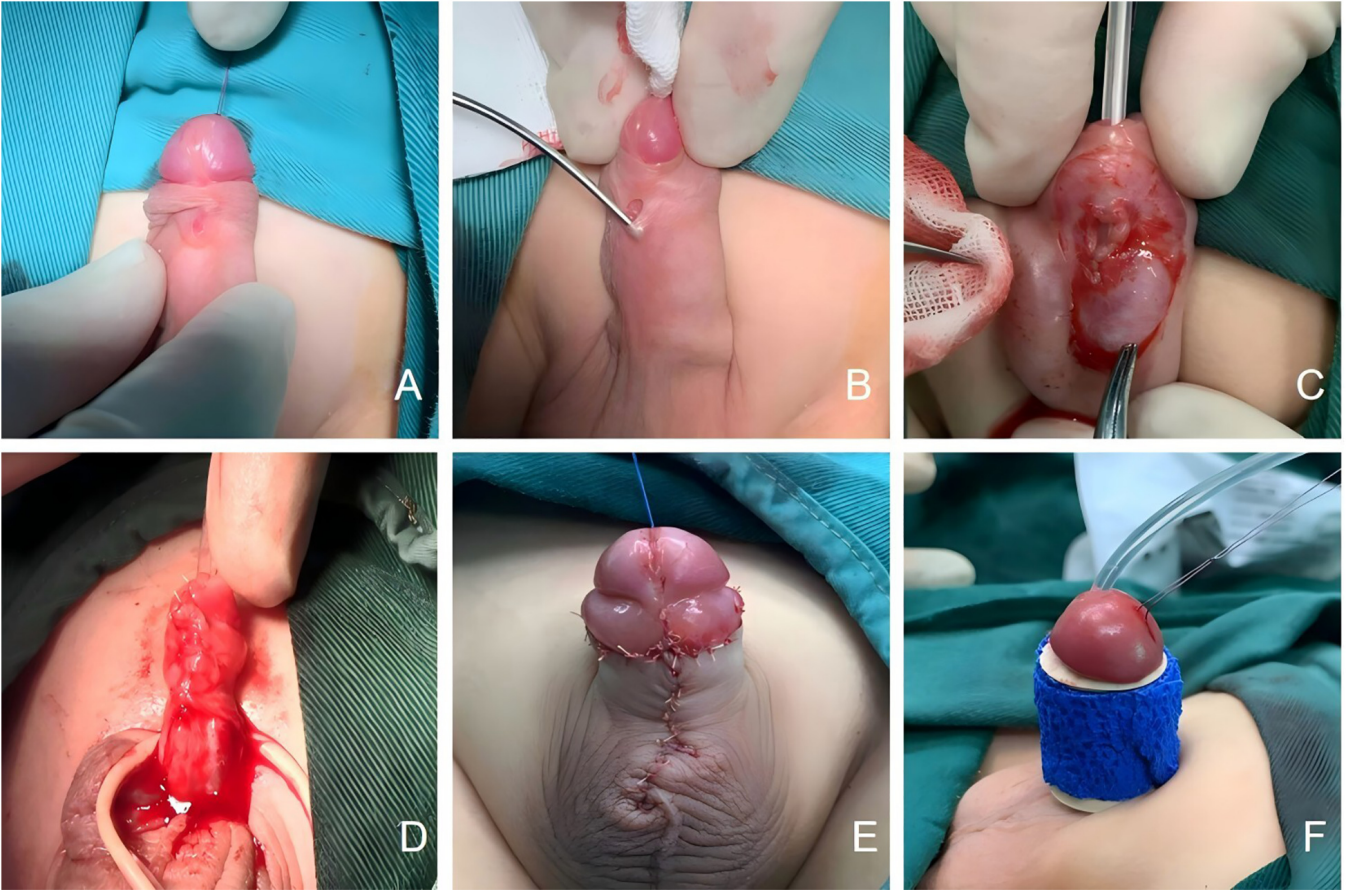
**(A)** The fistula of Congenital anterior urethrocutaneous fistula is located at the mid-penile anterior shaft; **(B)** a dysplastic membranous urethra is visible around the fistula; **(C)** suturing the urethral plate to complete the urethral repair; **(D)** use surrounding fascia to cover the urethra; **(E)** reshape the appearance of the penis; **(F)** dressing and bandaging the penis.

Seven patients underwent primary repair. In Cases 2 and 8, the fistula was located at the coronal sulcus; both patients had well-developed glans and sufficient ventral subcutaneous tissue, making them suitable candidates for the Mathieu technique. Cases 1, 3, and 5, who exhibited no penile curvature and had well-developed urethral plates with sufficient surrounding soft tissue, were treated with the Duplay technique.In Case 1, the fistula was located on the anterior midshaft of the penis (Figure 1A), with a patent distal urethra capable of accommodating an F8 catheter. A membranous urethra of underdeveloped tissue surrounded the fistula (Figure 1B), and the urethral meatus was normally positioned and shaped. An incision was made around the fistula, the urethral plate was mobilized by releasing the surrounding fascia, and reconstruction was achieved using a catheter as a stent. The urethral plate was sutured with 6-0 absorbable sutures to complete the repair (Figure 1C). The surrounding fascial tissue was mobilized to cover the repaired urethra (Figure 1D), the preputial appearance was reshaped (Figure 1E), and the penis was dressed (Figure 1F). For Cases 2 and 8, with fistulas at the coronal sulcus, and for Cases 3 and 5, with midshaft fistulas, the urethral repairs were covered with periurethral fascial tissue.

In Case 4, the urethra was mobilized, and preputial degloving was performed. An artificial erection test revealed a 30° ventral curvature. The dorsal neurovascular bundle was carefully dissected and preserved, and dorsal tunica albuginea plication was performed to correct the curvature. Due to the narrowness of the urethral plate, it was unable to accommodate an F8 catheter. Therefore, a midline incision was made along the urethral plate to the tunica albuginea to widen the channel. The repair was completed using the TIP method, with scrotal dartos tissue utilized to cover the urethra.

In Case 7, the fistula was located below the coronal sulcus. The urethral plate was narrow, and an artificial erection test revealed a 25° penile curvature. The fibrous penile bands were released, resulting in satisfactory straightening of the penis, and the repair was performed using the Onlay method.

In Case 6, the fistula was located at the penoscrotal junction. During surgery, the penile skin was degloved, and the fistula was mobilized. A long segment of membranous urethra, approximately 2.5 cm in length, was identified distally and incised. An artificial erection test revealed a 35° penile curvature, so the urethral plate was transected to correct the curvature. The fistula site was shifted proximally, resulting in a 3.5 cm urethral defect. The repair was carried out using the Duckett method, with a pedicled inner preputial flap wrapped around an F8 catheter and continuously sutured to form a new urethral tube. The distal end of the neourethra was positioned at the glans, and a proximal urethrostomy was created. A secondary procedure for urethrostomy closure was performed 10 months later. The mean duration of the first-stage surgery for all 8 patients was 100.75 ± 27.88 min.

All eight patients underwent successful surgical procedures, with preservation of the original external urethral meatus. For Patient 6, the initial one-stage repair showed satisfactory recovery, and a second-stage procedure for stoma closure was performed 10 months later. The median duration of urinary catheter placement postoperatively was 13 days (range: 12–14 days). Postoperatively, all patients demonstrated well-healed penile skin without complications such as bleeding or wound infections. During a follow-up period of 15–154 months, all patients achieved normal voiding, with no recurrence of urethrocutaneous fistula, urethral stricture, urethral diverticulum, or penile curvature. Based on informal post-operative interviews, 7 out of 8 parents of the children expressed high satisfaction with the surgical outcome, while 1 parent expressed general satisfaction. Given the satisfactory clinical outcomes observed, no instrumental follow-up assessments (e.g., uroflowmetry or VCUG) were performed.

## 4 Discussion

Literature reports indicate that the most common location for the fistula is the coronal sulcus (46%–53%), followed by the midshaft of the penis (approximately 38%) (2, 10). In this study, 3 of the 8 patients (37.5%) had fistulas at the coronal sulcus, and 3 (37.5%) had them at the midshaft, which is consistent with previously reported data.

Caldamone et al. (4) reported the largest series of 14 cases of congenital anterior urethrocutaneous fistula, classifying them into simple and hypospadias-associated types. In the present study, 5 cases (62.5%) were of the simple type, while 3 cases (37.5%) were hypospadias-associated.

The exact pathogenesis of congenital anterior urethrocutaneous fistula remains unclear (4, 8, 11, 12). Goldstein (13), Olbourne (14), Ritchey (15), and Islam (16) suggested that insufficient testosterone secretion during fetal development leads to failure of the fusion of the distal urogenital folds or stunted development of the distal urethra. Even if the urethra forms, it may fail to induce coverage by the penile fascia or corpus spongiosum. Minor external trauma or infection can then lead to fistula formation. Caldamone et al. (4) proposed that the formation of distal fistulas might be caused by developmental discordance between the glans and the corpus spongiosum. Bhattacharya (12) and Alhazmi et al. (8) believed that the failure of the penile urethra at the glans to align with the penile urethra is the primary cause of coronal sulcus fistulas. Coplen et al. (17) reported a case of a prenatal diagnosis of a pre-urethral diverticulum, which ruptured after birth. This is similar to Case 5 in this study. Some scholars have suggested that this condition shares a pathophysiological mechanism with hypospadias (4, 8, 11). Bhattacharya (12) argued that congenital anterior urethrocutaneous fistula associated with hypospadias is essentially hypospadias, where the anterior urethra is not a true urethra but a fragile, non-cavernous, skin-like tube, which is prone to rupture and fistula formation.

As both the urogenital sinus and the primordial anorectum develop from the cloaca, anorectal malformations, represented by imperforate anus, are the most commonly associated extraurological anomalies in congenital anterior urethrocutaneous fistula. The reported incidence ranges from 13.7%–21.4% (2, 4, 10). Patient history and physical examination are critical for diagnosing congenital anterior urethrocutaneous fistula, and complementary imaging studies such as ultrasound, cystourethrography, and cystoscopy are helpful in identifying any associated urinary tract anomalies (1, 10). We recommend that for complex cases with concomitant anorectal malformations, routine investigations should include VCUG, cystourethroscopy, abdominal ultrasonography, and measurement of post-void residual urine. For patients without anorectal malformations, if normal voiding is observed, VCUG and cystourethroscopy are not necessary. In cases of Y-shaped urethral duplication, the accessory urethra opening at the proximal penis may be misdiagnosed as a congenital anterior urethrocutaneous fistula, while the main urethra opens distally at the glans, often underdeveloped or even obstructed (4, 18). Preoperative exploration of the external urethral meatus and fistula using a catheter can aid in the correct diagnosis.

Surgical management should be individualized based on the location and size of the fistula, the condition of the urethra, and the presence of any associated anomalies (19). For patients with the simple type, if the distal urethra and corpus spongiosum are normal, repair may be achieved using Duplay or Mathieu techniques (4, 6). In cases with underdeveloped urethra or corpus spongiosum, hypospadias repair techniques should be considered (3, 4). Hypospadias-associated cases should be repaired based on the degree of penile curvature and urethral condition, with options including Duplay, Denis-Brown, Onlay, or TIP techniques (4, 10). For cases with severe penile curvature and poor urethral development, Duckett's method, free flaps, or staged surgery should be considered (4, 12). In this study, Cases 1, 3, and 5 were of the simple type, with adequate skin tissue surrounding the fistula, and were successfully repaired using the Duplay method. Cases 2 and 8, with fistulas located at the coronal sulcus and well-developed glans, were successfully repaired using the Mathieu technique. In Case 6, despite a small fistula (0.6 cm), the long segment of membranous urethra and the associated urethral tension were contributing factors to penile curvature. Therefore, membranous urethra was excised, and the distal urethral plate was transected to correct the curvature. The distal portion of the fistula remained intact, and the repair was completed using Duckett's method, with an anastomosis to the glans urethra. A secondary procedure for urethrostomy closure was performed later.

Soft tissue coverage and deep burial of the urethra are key strategies for preventing fistula recurrence (5, 10, 20). For small fistulas, fascial coverage may be sufficient (8), while larger fistulas benefit from the use of pedicled fascial flaps to prevent recurrence (1, 10). The primary coverage materials used in this study were periurethral fascia or dartos fascia, and the surgical outcomes were satisfactory. We recommend that the surgical approach prioritize adjacent, easily accessible, and tissue-rich soft tissues. If the surrounding tissue around the fistula is insufficient, dartos fascia, tunica vaginalis, or dorsal preputial fascia can be used. For cases requiring simultaneous correction of scrotal cleft, using dartos fascia for coverage is a more suitable option. Overall, congenital anterior urethrocutaneous fistula has a favorable treatment outcome, with success rates exceeding 90% (8, 10). However, complex cases, such as those involving anorectal malformations, present greater surgical challenges and longer treatment durations. Raj et al. (21) reported a case of a complex fistula associated with anorectal atresia, V-type short bowel syndrome, and Meconium peritonitis, which involved multiple organ systems and was challenging to treat.

Based on our hospital's cases and a review of the literature, we propose the following conclusions: (1) In patients without associated anorectal malformations, if they can void normally, cystourethrography and urethroscopy may not be required. (2) For cases with underdeveloped distal urethra or corpus spongiosum, even in simple cases, surgical intervention should be performed, and hypospadias repair techniques should be used. If the fistula is located near the external urethral meatus or the intervening tissue is too thin, repair using hypospadias techniques should also be considered. (3) Regardless of fistula type, soft tissue coverage and deep burial of the urethra should be performed. (4) Hypospadias-associated cases should be managed by experienced specialists using the most appropriate technique for the specific case. (5) Complex cases with significant anorectal malformations require careful preoperative evaluation of the anatomical relationships between the penis, urethra, and gastrointestinal tract, and multidisciplinary collaboration may be necessary for optimal management.

## 5 Conclusion

The diagnosis of congenital urethral cutaneous fistula requires careful examination to rule out the presence of other associated malformations. This helps in selecting the optimal surgical approach, thereby increasing the success rate of the surgery and reducing postoperative complications. This study is limited by its retrospective design and small sample size, which restricts statistical power and limits the generalizability of the findings. Further prospective studies with larger cohorts are necessary to corroborate these results. Additionally, as a single-center study, our results may reflect institutional preferences and patient referral patterns, which could limit broader applicability. How to choose the appropriate surgical method based on the patient's specific condition, as well as the long-term recovery outcomes post-surgery, still requires determination through larger-scale randomized trials and longer follow-up periods.

## Data Availability

The original contributions presented in the study are included in the article/Supplementary Material, further inquiries can be directed to the corresponding author.
